# A new metabolic gene signature in prostate cancer regulated by JMJD3 and EZH2

**DOI:** 10.18632/oncotarget.25182

**Published:** 2018-05-04

**Authors:** Marine Daures, Mouhamed Idrissou, Gaëlle Judes, Khaldoun Rifaï, Frédérique Penault-Llorca, Yves-Jean Bignon, Laurent Guy, Dominique Bernard-Gallon

**Affiliations:** ^1^ Department of Oncogenetics, Center Jean Perrin, CBRV, 63001 Clermont-Ferrand, France; ^2^ INSERM U1240, IMoST, University Clermont Auvergne, 63005 Clermont-Ferrand, France; ^3^ Department of Biopathology, Center Jean Perrin, 63011 Clermont-Ferrand, France; ^4^ Department of Urology, CHU Gabriel Montpied, 63000 Clermont-Ferrand, France

**Keywords:** prostate cancer, JMJD3, EZH2, gene panel, epidrugs

## Abstract

Histone methylation is essential for gene expression control. Trimethylated lysine 27 of histone 3 (H3K27me3) is controlled by the balance between the activities of JMJD3 demethylase and EZH2 methyltransferase. This epigenetic mark has been shown to be deregulated in prostate cancer, and evidence shows H3K27me3 enrichment on gene promoters in prostate cancer.

To study the impact of this enrichment, a transcriptomic analysis with TaqMan Low Density Array (TLDA) of several genes was studied on prostate biopsies divided into three clinical grades: normal (*n* = 23) and two tumor groups that differed in their aggressiveness (Gleason score ≤ 7 (*n* = 20) and >7 (*n* = 19)). ANOVA demonstrated that expression of the gene set was upregulated in tumors and correlated with Gleason score, thus discriminating between the three clinical groups. Six genes involved in key cellular processes stood out: *JMJD3*, *EZH2*, *MGMT*, *TRA2A*, *U2AF1* and *RPS6KA2*. Chromatin immunoprecipitation demonstrated collocation of EZH2 and JMJD3 on gene promoters that was dependent on disease stage. Gene set expression was also evaluated on prostate cancer cell lines (DU 145, PC-3 and LNCaP) treated with an inhibitor of JMJD3 (GSK-J4) or EZH2 (DZNeP) to study their involvement in gene regulation. Results showed a difference in GSK-J4 sensitivity under PTEN status of cell lines and an opposite gene expression profile according to androgen status of cells.

In summary, our data describe the impacts of JMJD3 and EZH2 on a new gene signature involved in prostate cancer that may help identify diagnostic and therapeutic targets in prostate cancer.

## INTRODUCTION

Prostate cancer is the most common male cancer in developed countries, with 758,700 estimated new cases and 307,500 estimated deaths in 2012 [1]. The disease is multifactorial, and includes genetic and environmental risk factors [2, 3]. Moreover, prostate cancer is strongly linked to epigenetic alterations resulting in aberrant gene expression [4], particularly with histone methylation, which defines chromatin structure and accessibility to transcription factors [5, 6].

Trimethylated lysine 27 of histone 3 (H3K27me3) is a repressive epigenetic mark, and studies show that an aberrant level of it in prostate cancer leads to dysregulation on gene expression [7–9]. H3K27me3 levels are determined by histone methyltransferase EZH2 (enhancer of zeste homolog 2) and histone demethylase JMJD3 (jumonji domain-containing 3), and both these proteins are upregulated in prostate cancer [10]. Control of this mark therefore plays a key role in cell integrity, and is a potential biomarker for prostate cancer.

EZH2, which is the catalytic subunit of polycomb repressive complex 2 (PRC2), plays a predominant role in various cellular processes such as cell cycle regulation and proliferation [11, 12]. This widely studied protein is shown to have all the oncogene properties, its overexpression stimulating cell proliferation and invasion, but it is also reported in solid prostate malignancies [13, 14]. Moreover, EZH2 has coactivator functions of a transcription factor by polycomb-independent activity in castration-resistant prostate cancer cells [15].

The first EZH2 inhibitor is DZNeP (3-deazaneplanocin A) and has antitumor activity [16]. This drug inhibits *S*-adenosylhomocysteine hydrolase (SAH), which causes indirect repression of *S*-adenosylmethionine (SAM)-dependent histone lysine methyltransferase. Injection of prostate cancer cells pre-treated with DZNeP in male immunocompromised NOD/SCID mice induced a reduction of tumor formation in LNCaP and inhibits tumor growth in DU 145 [17]. Furthermore, their treatment with DZNeP shows re-expression of H3K27me3-enriched genes (*RARβ2*, *ERα*, *RGMA* and *PGR*) [9]. These reversible effects are attractive targets for a therapeutic approach.

Histone demethylases are epigenetic actors with a crucial role in cancer by acting as suppressors of tumors or as oncogenes [18]. JMJD3 and UTX (ubiquitously transcribed tetratricopeptide repeat, X chromosome) are transcription activators, being specific H3K27me3 demethylases. JMJD3 is involved in many cellular process such as development, differentiation, senescence and aging by p16, p53 and RB pathways and finally inflammation [19]. Depending on cancer type, JMJD3 expression is increased (prostate and breast cancers, melanoma, gliomas, renal cell carcinoma [10, 20–24]) or decreased (lung, liver, pancreatic, colon and colorectal cancers [25–27]). This role in carcinogenesis has allowed the development of “epidrugs” to modulate JMJD3 expression.

Several studies have shown that JMJD3 depletion by GSK-J4 chemical inhibitor, an ethyl ester derivative of GSK-J1, could offer a new therapeutic approach in various diseases [28–30], and highlight its anti-tumor activities on brainstem gliomas and breast cancer stem cells in xenograft models [31, 32].

A previous study by genome-wide microarrays reported differentially H3K27me3-enriched regions in prostate cancer [33]. To understand molecular mechanisms of H3K27me3 enrichment and identify new potential gene targets in prostate cancer for improved prognosis and diagnosis, we performed transcriptomic analysis on a TaqMan Low Density Array (TLDA) of selected genes in prostate tissues. We also investigated the impact of EZH2 and JMJD3. First, we performed ChIP-qPCR with JMJD3 and EZH2 antibodies to identify occupancy of both these proteins on gene promoters. Secondly, we identified their effects on gene expressions after pharmacologic inhibition with DZNeP or GSK-J4 treatments.

## RESULTS

### Gene set expression is increased in prostate cancer

To investigate the impact of H3K27me3 enrichment on gene expression [33], and identify potential new actors in prostate cancer, we performed transcriptomic analysis using TaqMan Low Density Array (TLDA) technology on 23 selected genes (Table 1). Gene expression was explored in prostate biopsies representing three clinicopathological groups: a normal group (*n* = 23) and two tumor groups classified according to tumor aggressiveness by their Gleason score (GS): GS ≤ 7 (*n* = 20) and GS > 7 (*n* = 19).

**Table 1:** Gene list designed on TaqMan Low Density Array

Gene symbol	Assay reference	Gene name
NMNAT2	Hs00322752_m1	Nicotinamide nucleotide adenylyltransferase 2
ESRRG	Hs00976243_m1	Estrogen-related receptor gamma
CDH20	Hs00230412_m1	Cadherin 20
KDM6B	Hs00996325_g1	Lysine demethylase 6B
ING3	Hs00219444_m1	Inhibitor of growth family member 3
RXRG	Hs00199455_m1	Retinoic acid receptor gamma
WT1-AS	Hs00274809_s1	Wilms tumor 1 antisense RNA
PPP2R5E	Hs00952135_m1	Protein phosphatase 2 regulatory subunit B’epsilon
EZH2	Hs00544833_m1	Enhancer of zeste homolog 2
IRX1	Hs00411782_m1	Iroquois homeobox 1
18S	Hs99999901_s1	-
TRIM40	Hs00373297_m1	Tripartite motif containing 40
MGMT	Hs01037698_m1	*O*-6-Methylguanine-DNA methyltransferase
PAPOLG	Hs00224661_m1	Poly(A) polymerase gamma
U2AF1	Hs01597465_g1	U2 small nuclear RNA auxiliary factor 1
TRA2A	Hs00203263_m1	Transformer 2 alpha homolog
RPS6KA2	Hs00179731_m1	Ribosomal protein S6 kinase A2
PIK3CB	Hs00927728_m1	Phosphatidylinositol-4,5-bisphosphate 3-kinase catalytic subunit beta
SGK1	Hs00985033_g1	Serum/glucocorticoid regulated kinase 1
KDR	Hs00911700_m1	Kinase insert domain receptor
SGMS1	Hs00983630_m1	Sphingomyelin synthase 1
TMPRSS6	Hs00542184_m1	Transmembrane protease, serine 6
SLC4A4	Hs00186798_m1	Solute carrier family 4 member 4
CNNM2	Hs00929652_m1	Cyclin and CBS domain divalent metal cation transport mediator 2

**Figure 1:**
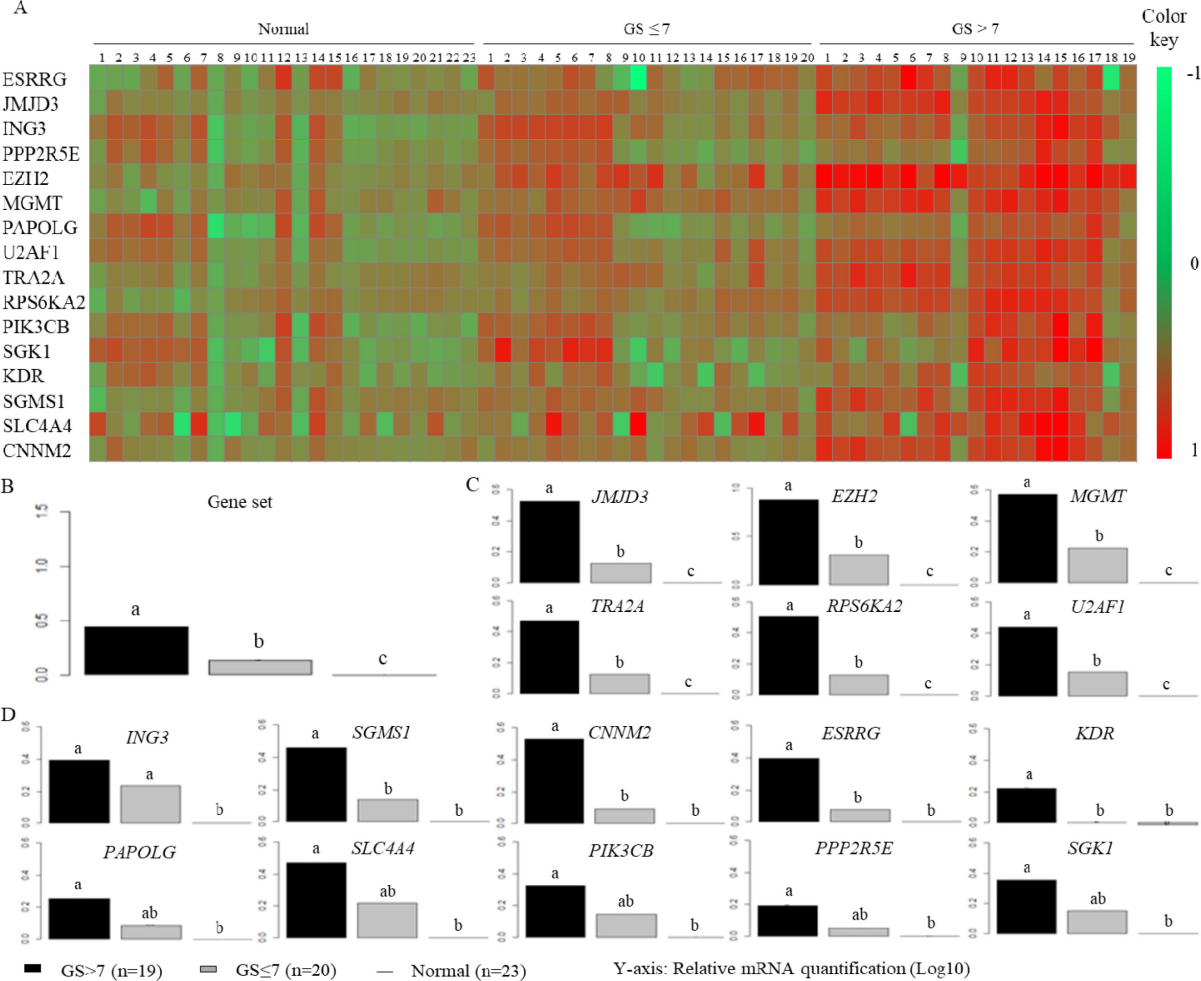
Assessment of gene mRNA expression between clinicopathological groups mRNA expression was obtained using RT-qPCR by TaqMan Low Density Array (TLDA) in normal tissues (*n* = 23, line) and tumors with different GS: GS ≤ 7 (*n* = 20, gray bars) and GS > 7 (*n* = 19 black bars). Representation is the relative mRNA quantification in Log10, and 18S RNA was used as an internal control in the PCR reaction. (**A**) Representation in heat map format of gene expression. Row represents a gene and column represents clinic groups, each column represents one patient and is illustrated according to a color scale from green to red. (**B**–**D**) ANOVA analysis of gene mRNA expression; *y*-axis corresponds to relative mRNA quantification. An analysis of variance followed by a Tukey multiple comparison test designates the statistically significant variables by the letters a, b and c.

A heat map representation of RT-qPCR shows increased gene expressions in patients with cancer compared with healthy patients, and related to higher GS (Figure 1A). We thus observed clinicopathological group compartmentalization according to the gene expressions.

To confirm this observation, we performed an analysis of variance (Figure 1B–1D). Figure 1B shows that gene set expression was significantly increased in tumor groups, contrasting with the normal group, and it correlated with GS. An elevated gene expression was observed, consistent with tumor aggressiveness. More precisely, we observed four different expression profiles, but in every case we noted a significant increase in transcriptional expression in tumors with higher GS compared with normal tissues (Figure 1C–1D). The first profile is the most interesting one because gene expression significantly discriminated between the three clinical groups: it includes six genes: *JMJD3*, *EZH2*, *MGMT*, *TRA2A*, *RPS6KA2* and *U2AF1* (Figure 1C). Figure 1D shows that *ING3* expression discriminated both tumor groups compared with the normal group. The third profile distinguished tumors with GS > 7 from normal tissues, and distinguished intermediate grade tumors (GS ≤ 7) for *PAPOLG*, *SLC4A4*, *PIK3CB*, *PPP2R5E* and *SGK1*. Finally, *SGMS1*, *CNNM2*, *ESRRG* and *KDR* expressions discriminated tumors with poor clinical prognosis compared with other grades.

**Figure 2:**
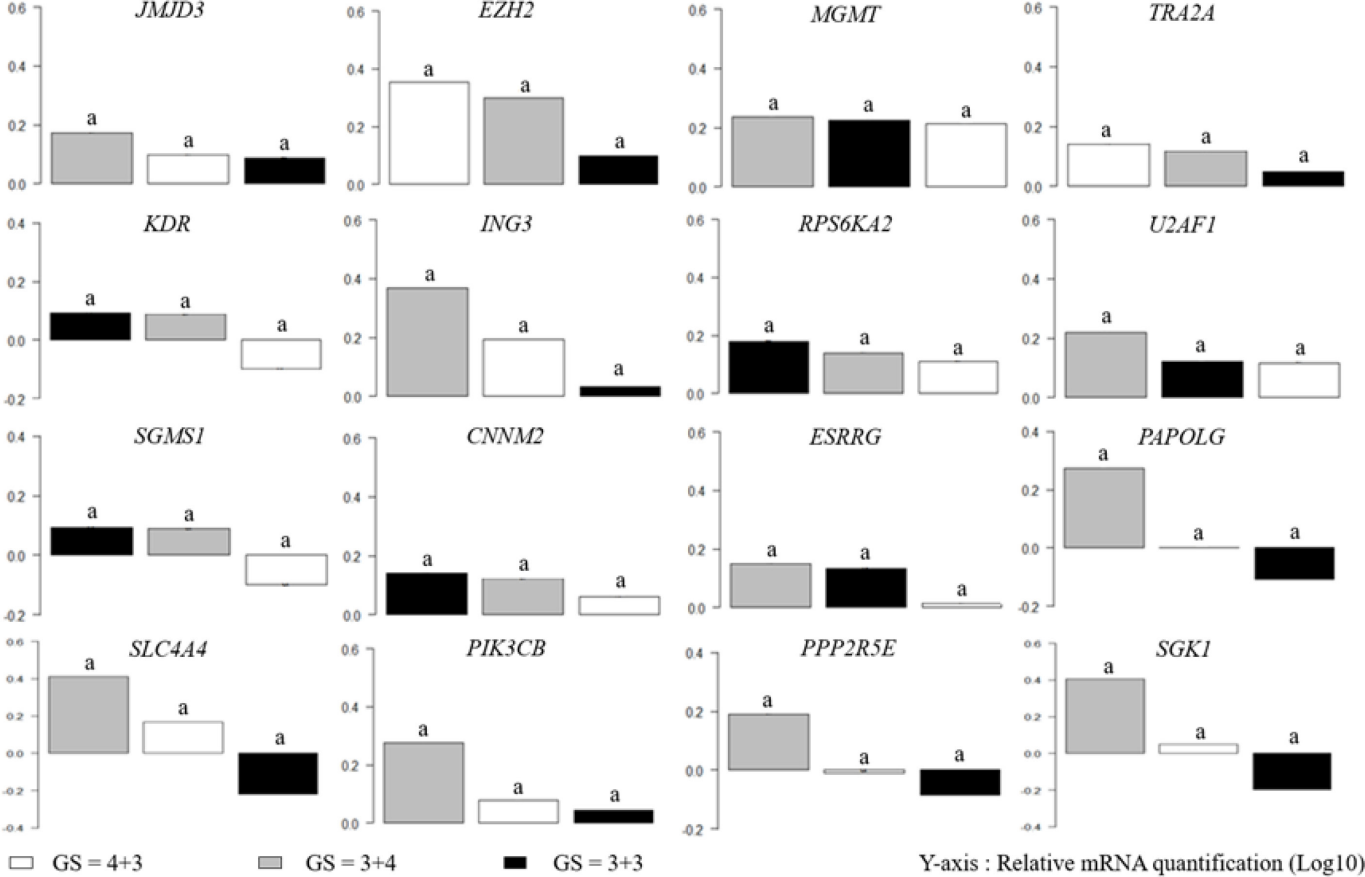
mRNA expression is not significantly different in GS ≤ 7 clinicopathological groups ANOVA analysis was performed on prostate biopsies with GS ≤ 7 (GS = 4+3 (*n* = 11, white bars), GS = 3+4 (*n* = 7, gray bars) and GS = 3+3 (*n* = 2, black bars)); *y*-axis corresponds to relative mRNA quantification in Log10. An analysis of variance followed by a Tukey multiple comparison test was performed.

Recent studies developed a new set of prostate grade groups, splitting GS ≤ 7 into three grades: GS ≤ 6, GS = 3+4 and GS = 4+3 [34]. To validate our classification, we performed the same statistical analysis on group GS ≤ 7 split into three groups (Figure 2). Results show no significant difference between grades, and so validated our overall classification for the GS ≤ 7 group.

### Loss of gene expression control by JMJD3 and EZH2 in prostate cancer

To understand the clinical group discriminations by transcriptional expression of *MGMT*, *TRA2A*, *U2AF1* and *RPS6KA2* and the involvement of JMJD3 and EZH2 in their regulation, we performed a ChIP assay to study their collocation on prostate tissues with the same validated classification: normal (*n* = 12), GS ≤ 7 (*n* = 22) and GS > 7 (*n* = 6).

**Figure 3:**
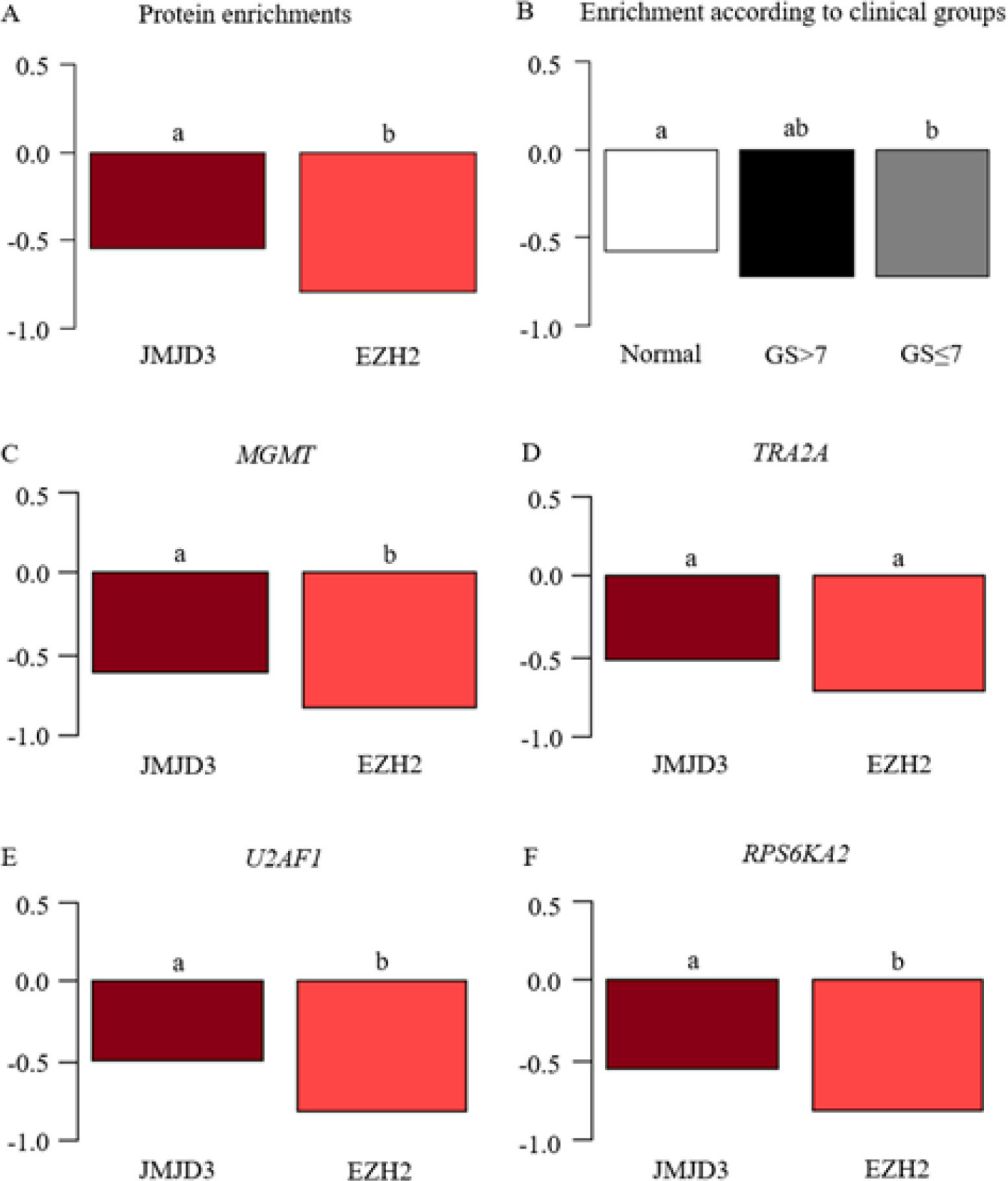
JMJD3 and EZH2 enrichment on *MGMT*, *TRA2A*, *U2AF1* and *RPS6KA2* promoters ChIP analysis studied the change of JMJD3 and EZH2 on four gene promoters (*MGMT*, *TRA2A*, *U2AF1* and *RPS6KA2*) in prostate tumor tissues (GS ≤ 7 (*n* = 22) and GS > 7 (*n* = 6)) compared with normal tissues (*n* = 12); *y*-axis corresponds to percentage of input in Log10. An analysis of variance followed by a Tukey multiple comparison test designated the statistically significant variables by the letters a and b. (**A**) EZH2-enrichment was lower compared to JMJD3 throughout promoter gene or clinical groups. (**B**) Interaction between proteins and genes depends on clinical groups. (**C**–**F**) ChIP analysis for individual gene.

We demonstrated that EZH2 occupancy on gene promoters was significantly less than JMJD3 irrespective of gene and clinical group (Figure 3A), evoking a possible lower activity of EZH2 compared with JMJD3. This observation supports gene expression results: a higher demethylation inducing transcriptional activation. In detail, if we observed gene expression one by one, this observation was also significantly found for *MGMT*, *U2AF1* and *RPS6KA2*, but we observed only a trend for *TRA2A* (Figure 3C–3F).

Furthermore, protein recovery was significantly lower in the intermediate grade (GS ≤ 7) compared with normal tissues, and the same trend was observed for the aggressive tumor group (GS > 7), suggesting a loss of gene expression control by JMJD3 and EZH2 (Figure 3B).

### Effects of GSK-J4 on cell viability

**Figure 4:**
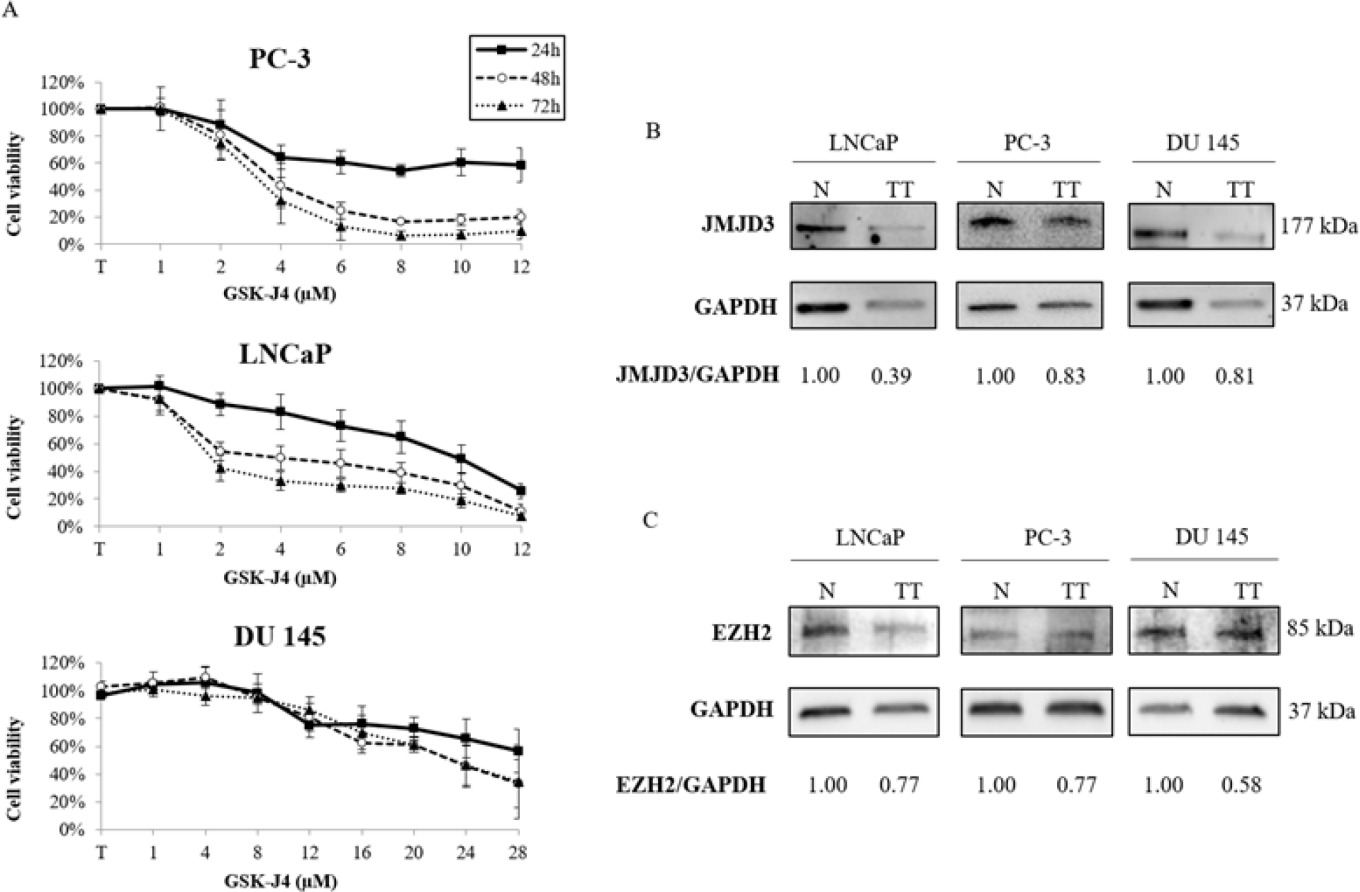
Effects of GSK-J4 or DZNeP on prostate cancer cell lines (**A**) Cell line viability treated with GSK-J4. PC-3, LNCaP and DU 145 cells were treated for 24 h, 48 h and 72 h with increasing concentrations of GSK-J4. Cell viability was assessed using the XTT assay. The percentage of viable cells was determined as percent of viability of untreated cells. Values shown are the average (mean ± S.E.M) from quadruplicate samples for each incubation condition. (**B**–**C**) Western-blot analysis of GSK-J4 and DZNeP efficacity. Cells were treated at IC_50_ concentration (PC-3: 3.53 μM, LNCaP: 3.93 μM and DU 145: 22.87 μM for 48 h for GSK-J4 and 10 μM for 72 h for DZNeP) (TT) or untreated (N). Quantification representation were expressed as relative fold change in protein expression of JMJD3 and EZH2 in response to GSK-J4 or DZNeP exposure respectively after normalization to GAPDH density.

To determine the cytotoxicity of GSK-J4, a chemical inhibitor of JMJD3, three prostate cancer cell lines, DU 145, PC-3 and LNCaP, were treated at increasing concentration for 24 h, 48 h and 72 h. Figure 4A shows a considerable concentration-dependent decrease in cell proliferation with treatment. An IC_50_ study of GSK-J4, exhibited a wide disparity in its concentration according to the cell line: the IC_50_ values of PC-3 and LNCaP were 3.53 μM and 3.93 μM respectively, and conversely was 22.87 μM for DU 145.

Finally, we performed a Western blot to verify treatment actions. JMJD3 expression with GSK-J4 treatment was reduced by 61% for LNCaP and by around 20% for DU 145 and PC-3 (Figure 4B). For DZNeP treatment, EZH2 was decreased by 42% for DU 145 and by around 25% for LNCaP and PC-3 (Figure 4C).

### Impact of JMJD3 and EZH2 on gene regulation

To highlight the involvement of methyltransferase and demethylase on gene regulation, we performed transcriptomic analysis on prostate cancer cell lines treated and untreated with their inhibitors (GSK-J4 or DZNeP).

**Figure 5:**
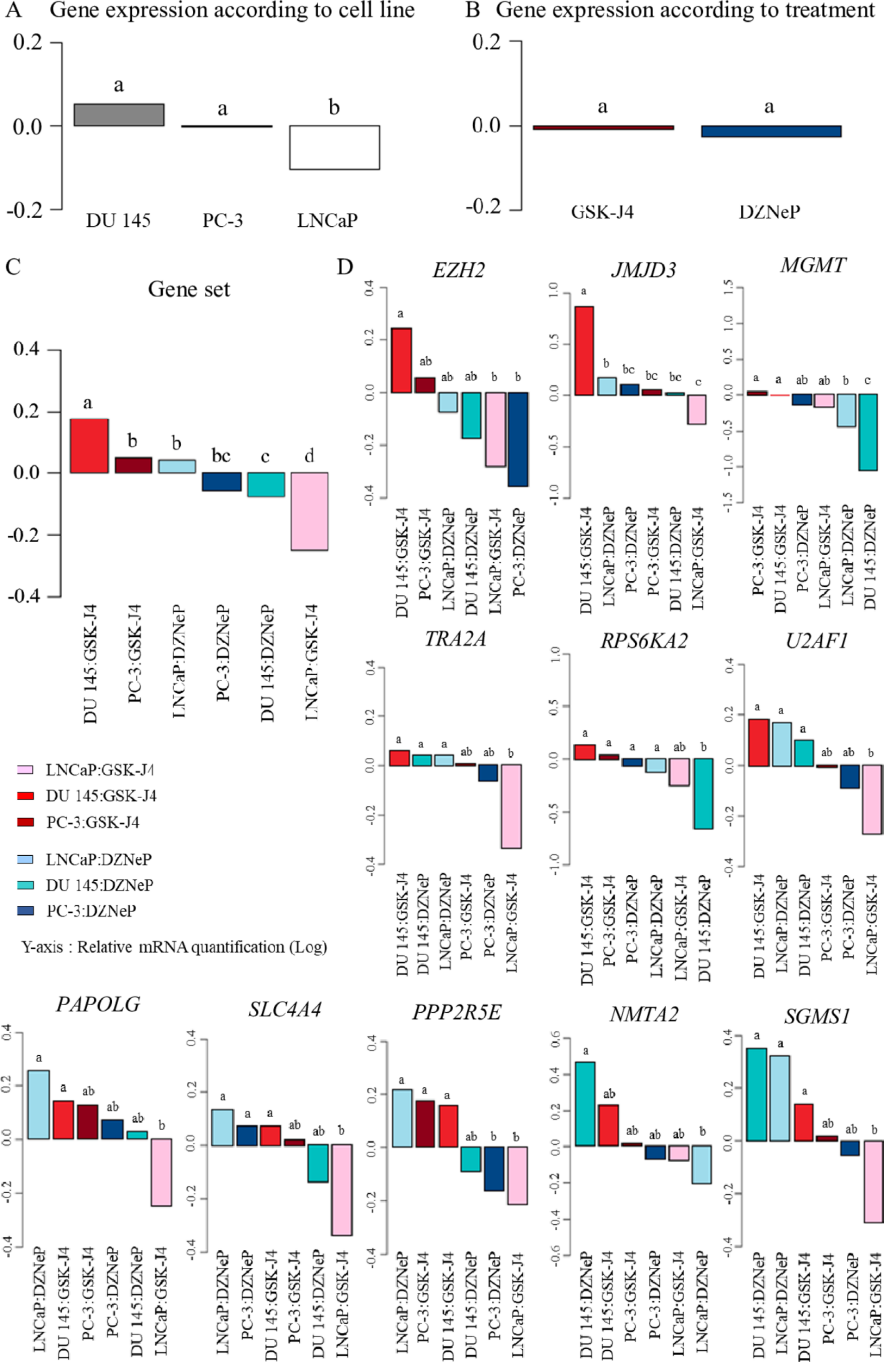
Effects of treatment and cell line on the gene set expression ANOVA analysis of gene mRNA expression was performed on cell lines (PC-3 (black bars), DU 145 (gray bars) and LNCaP (white bars)) treated with GSK-J4 (neutral bar) or DZNeP (striped bar). Values shown are the average (mean ± S.E.M) from quadruplicate samples for each incubation condition and normalized to control without treatment; *y*-axis corresponds to relative mRNA quantification in Log. Test designated the statistically significant variables by the letters a, b and c. (**A**) Gene set expression was different according to cell lines, but (**B**) not significant according to treatments. (**C**) Combined effect of cell lines and treatments on whole gene expression. (**D**) Combined effect of cell lines and treatments gene per gene.

Statistical analysis of the cell line effects independent of the treatments showed an opposite expression of the gene set in LNCaP compared with the other cell lines (Figure 5A–5B). Analysis of the combined effects of cell line and treatment also showed that gene set expression was upregulated in DU 145 and PC-3 treated with GSK-J4, contrary to LNCaP. We observed the reverse effect for DZNeP treatment (Figure 5C).

More precisely, Figure 5D showed the same profile of gene set expression compared with *EZH2* for GSK-J4 treatment by contrast with DZNeP treatment: EZH2 inhibitor treatment leads to a reduction of its expression, with a trend according to cell line aggressiveness, and demonstrated its impact on transcriptional level. *JMJD3* expression displays the same distinction between DU 145 and the other cell lines for GSK-J4 treatment, but DU 145 treated with GSK-J4 shows a marked increase in *JMJD3* expression.

## DISCUSSION

We began with a transcriptomic analysis on several H3K27me3-enriched genes in prostate cancer. We demonstrated that gene set expression was upregulated in tumors compared with normal tissues and in correlation with Gleason score (Figure 1). In particular, six genes emerged from this study: *JMJD3*, *EZH2*, *MGMT*, *TRA2A*, *RPS6KA2* and *U2AF1*.

MGMT is a DNA repair protein, and its DNA hypermethylation has been reported in human cancers [35]. However, a discrepancy in prostate cancer was found. On the one hand, *MGMT* was shown to be hypermethylated in prostate cancer according to androgen sensitivity and cause a loss of expression [36], but on the other hand, Maruyama *et al.* found no methylation on this gene, coming closer to our results [37]. A possible explanation of this opposition may lie in ethnic and environmental factors and the disease stage: a meta-analysis in gastric cancer shows heterogeneity on *MGMT* methylation between Asian and Caucasian populations [38].

Our results support the emergent role of the spliceosome pathway in prostate carcinogenesis [39] with TRA2A and U2AF1: TRA2A is deregulated in different cancers such as hepatocellular carcinoma, pediatric pineal germinomas and triple-negative breast cancer (TNBC) [40–42]. Deregulation of U2AF1 is observed in lung carcinoma [43], and it is mutated in myelodysplastic syndrome [44].

RPS6KA2 (or RSK3), belonging to the RSK (ribosomal S6 kinase) family, is a downstream effector of the Ras/MAPK pathway. Many studies show that deregulation of RSK proteins is associated with cancer development [45], but isoforms have opposite functions: RSK1 and RSK2 are considered as oncogenes, proved in many cancers (breast, lung, leukemia) including prostate, with RSK2-mediated increase in PSA expressions [46] compared with RSK3 and RSK4, but RSK3 activity has been studied only in ovarian and breast cancers [47, 48], and is unknown in prostate tumorigenesis.

Our gene set analysis thus discriminates between the three clinicopathological groups, and highlights genes involved in key cellular processes of carcinogenesis.

We went on to investigate the involvement of JMJD3 and EZH2 on gene regulation. First, we determined the collocation of JMJD3 and EZH2 on *MGMT*, *TRA2A*, *RPS6KA2* and *U2AF1* promoters suggesting a control of both proteins on their gene regulation (Figure 3). Specifically, we identified a lower recovery of EZH2 compared with JMJD3 implying a greater activity of the latter, and so a better demethylation of H3K27me3, and a transcriptional activation of target genes. Other studies had shown greater recovery of JMJD3 in contrast to EZH2 on H3K27me3-enrichment genes in prostate cell lines [10] and in prostate tissues [49]. These findings confirm the importance of EZH2 and particularly JMJD3 in gene regulation in prostate cancer.

**Figure 6:**
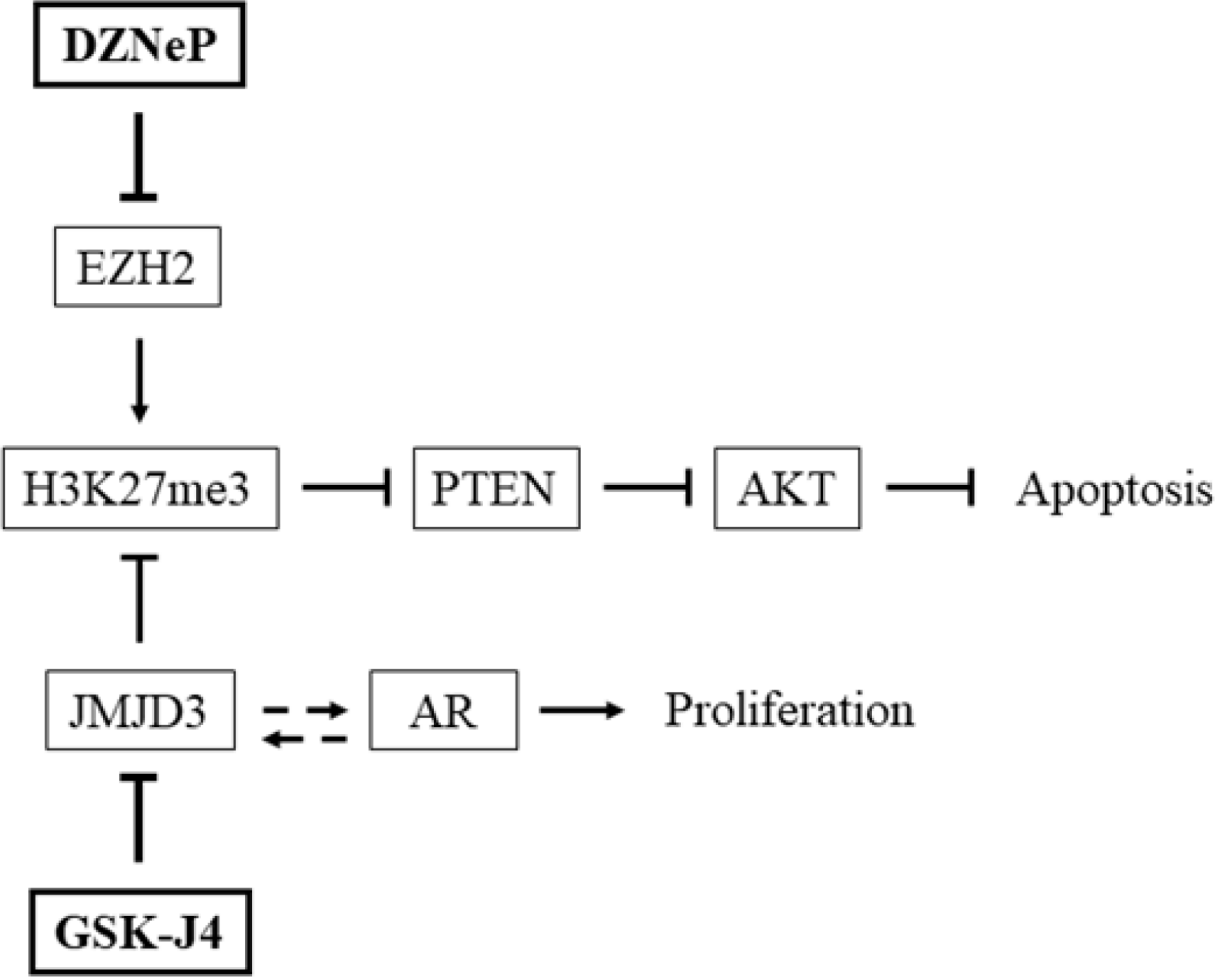
Interaction of GSK-J4 and DZNeP on PTEN and AR pathways Effects of JMJD3 and EZH2 inhibitors on key pathways involved in prostate cancer. JMJD3 inhibitor GSK-J4 enhanced H3K27me3 which inhibited PTEN expression and activated AKT; by contrast, DZNeP counteracted these effects. GSK-J4 acts on AR-driven transcription and interferes with proliferation. Arrows indicate an activation, blocked arrows indicate an inhibition and dotted arrows a presumed interaction.

We tested the impact of GSK-J4 and DZNeP, chemical inhibitors of JMJD3 and EZH2 respectively on prostate cancer cell lines. Firstly, we observed a difference in treatment responses with GSK-J4 between DU 145 and other cell lines, PC-3 and LNCaP (Figure 4A). The IC_50_ was around six times higher for DU 145 (22.87 μM) compared with PC-3 (3.53 μM) and LNCaP (3.93 μM). To explain this disparity, the distinction between cell lines was examined. We noted that LNCaP and PC-3 contained a constitutive AKT activity due to an inactivation or loss of PTEN function compared with DU 145, which expressed a functional PTEN protein [50]. Therefore, disparity of GSK-J4 concentration could therefore be explained by their difference in PTEN status, suggesting a possible link between PTEN activity and GSK-J4, and subsequently with JMJD3. PTEN is a tumor suppressor gene involved in the PI3K/AKT pathway, and is inactivated in several cancers including prostate [51]. Interplay between H3K27me3, EZH2 and PTEN is known [52, 53]; H3K27me3 targets and blocks *PTEN* transcriptional activation. By contrast, only one recent study shows interaction between GSK-J4/JMJD3 and this pathway; GSK-J4 treatment hindered H3K27me3 demethylation, leading to PTEN down-regulation in human monocytic cells [54]. Moreover, the sensitivity difference of GSK-J4 treatment was also observed in correlation with other key pathways of prostate tumorigenesis in castration-resistant prostate cancer cell lines compared with AR-WT prostate cancer cells, suggesting a AR-dependent involvement of JMJD3 [55]. The interplay between JMJD3 and the AR pathway was also evidenced in another study where the transcriptional level of JMJD3 was increased in LNCaP, which are AR-positive, compared with normal cells (PWR-1E) and AR-negative cells (PC-3) [10]. Our study supports this point, transcriptomic analysis in cells showing an opposition in gene expression in LNCaP (AR-positive) compared with both AR-negative cell lines, PC-3 and DU 145 (Figure 5A, 5C). In the light of these observations, GSK-J4 and DZNeP may be involved in key pathways, PTEN and AR, involved in prostate cancer (Figure 6).

In conclusion, we have identified a gene set modulated by JMJD3 and EZH2. These genes are key components in metabolic pathways involved in prostate cancer, and could be used as potential new biomarkers of prognosis, and also of aggressiveness in prostate cancer. Additionally, the use of epidrug GSK-J4 and DZNeP aimed at demethylase and methyltransferase might enable a new therapeutic strategy to be developed. An *in vivo* study is now needed to evaluate the impact of treatments on tumor growth, together with gene expression by a xenograft approach like that of Hashizume *et al.*, which demonstrated antitumor activity of GSK-J4 on pediatric brainstem glioma [31].

## MATERIALS AND METHODS

### Biopsy collection

Prostate biopsies were obtained from 62 patients for TLDA analysis and 40 patients for ChIP analysis diagnosed by a pathologist at Clermont-Ferrand University Hospital (France). All biopsies were kept in nitrogen. Patients did not receive chemotherapy before clinical examination. All subjects gave written informed consent to the study.

### Cell lines and culture conditions

DU 145, LNCaP and PC-3 were obtained from American Type Culture Collection (ATCC, Manassas, VA, USA) and conserved in liquid nitrogen at the Biological Resource, Jean Perrin Centre. Cells were cultivated in Eagle's minimum essential medium (EMEM) for DU 145 (ATCC), in RPMI 1640 medium for LNCaP (Gibco, Grand Island, NY, USA) and F-12K medium for PC-3 (ATCC). Cultures were supplemented with 10% heat-inactivated fetal bovine serum (FBS) (Life Technologies, Carlsbad, CA, USA), 1% glutamine and 0.1% gentamicin (Panpharma, Luitré, France). Cells were maintained in a monolayer culture at 37° C in a humidified atmosphere of 95% air and 5% CO_2_.

### Cell viability assay

Viability assays were performed with an XTT Cell Viability Kit (Biotium, Hayward, CA, USA) according to the manufacturer's protocol. 5000 cells were seeded in sixplicate, and treated with increased doses of GSK-J4 (Sigma-Aldrich, St Louis, MO, USA) for 24 h, 48 h or 72 h. After 2 h of XTT incubation, cell viability was determined by measuring the absorbance signal at 450 nm with a Multiskan™ GO Microplate Spectrophotometer (ThermoFisher Scientific). Viable cells were presented as a percentage of the untreated cell control, and IC_50_ was determined by linear interpolation between concentrations just above and below 50% inhibition in the response dose curve. IC_50_ was calculated using the formula: EXP(LN(conc > 50%)-((signal > 50%-50)/(signal > 50%-signal < 50%)xLN(conc > 50%/conc < 50%))).

### Western blotting

Total protein extractions were performed using RIPA buffer with 1% protease inhibitor and 1% phosphatase inhibitor cocktails (Sigma-Aldrich) according to the manufacturer's instructions. Protein concentrations were determined by the Bradford method (Bio-Rad Laboratories, Hercules, CA, USA). 35 μg of all protein samples were separated on 4–15% Mini-PROTEAN^®^ TGX™ Precast Protein Gels (Bio-Rad Laboratories) and transferred to nitrocellulose membranes (Bio-Rad Laboratories).

Membranes were blocked for 1 h at room temperature with 5% milk in TBST buffer (1X Tris-buffered saline, 0.1% Tween). Primary antibodies used were: anti-EZH2 (1:500, #C15410039, Diagenode, Seraing, Belgium) anti-JMJD3 (1:750, #ab169197, Abcam, Cambridge, UK) and anti-GAPDH (1:5000, #sc-25778, Santa Cruz Biotechnologies, Dallas, TX, USA). After three washes, membranes were blocked with secondary antibody anti-rabbit ads-HRP (1:5000, #4050-05, Southern Biotech, Birmingham, AL, USA).

Membranes were incubated in ECL Clarity Western Substrate (Bio-Rad Laboratories) and detection was performed on a ChemiDoc™ Touch Imaging System (Bio-Rad Laboratories) coupled with Image Lab™ Touch Software; quantification was expressed as the ratio of proteins over GAPDH densities.

### Quantitative real-time PCR

#### RNA extraction

Biopsies were disrupted in nitrogen solution with a French press. Total mRNA isolation was performed using an RNeasy Micro kit (Qiagen, Crawley, UK) according to the manufacturer's instructions, and RNA was eluted in 10 μl of RNase-free water.

For GSK-J4 treatment, cells were plated in T75 at a density of 1 × 10^6^ cells with IC_50_ concentration (PC-3: 3.53 μM, LNCaP: 3.93 μM and DU 145: 22.87 μM) and for DZNeP treatment cells were plated in 6-well plates at 0.5 × 10^5^ cells with 10 μM of DZNeP [9]. After 48 h of GSK-J4 treatment or 72 h of DZNeP treatment, cells were washed in phosphate-buffered saline (PBS, Life Technologies), and total mRNA isolation was performed using a TRIzol^®^ Plus RNA Purification kit (Invitrogen, Carlsbad, CA, USA) according to the manufacturer's protocol; RNA was eluted in 50 μL of RNase-free water.

Final RNA concentration and purity were measured using a NanoDrop ND-8000 spectrophotometer (NanoDrop Technology, LabTec).

#### Reverse transcription

1 μg of total mRNA per sample was reverse-transcribed in 20 μL total volume using a High Capacity cDNA Reverse Transcription kit (Applied Biosystems, Foster City, CA, USA) according to the manufacturer's instructions. Incubation was at 25° C for 10 min, reverse transcription was at 37° C for 120 min, and inactivation was at 85° C for 5 min.

#### qPCR using TaqMan Low-Density Array (TLDA)

24 gene expressions (Table 1) were quantified using a custom-made TLDA, which was a 384-well microfluid card (Applied Biosystems). This microfluid card can run 8 duplicate samples against 24 TaqMan Gene Expression Assay targets that are preloaded into each card well. 18S RNA was used as an internal control in the PCR reaction. 100 ng of cDNA was mixed with TaqMan Universal PCR Master Mix (Applied Biosystems). Samples were transferred into the sample-loading port of the TLDA and centrifuged twice for 1 min at 1200 rpm. TLDA was sealed to prevent contamination between wells. qPCR was performed, and cDNA was quantified with the TaqMan method (ABI Prism 7900 HT Sequence Detection System, Applied Biosystems) according to the manufacturer's instructions. Threshold cycle (Ct) higher than 35 as the threshold of non-expressed gene. The relative quantification (RQ) of gene expression was determined using the comparative ΔΔCt : RQ = 2^-ΔΔCt^ with ΔCt = Ct (target gene) – Ct (endogenous gene 18S) and ΔΔCt = ΔCt (tumoral group) - ΔCt (normal group) in patient study or ΔΔCt = ΔCt (treated cells) - ΔCt (untreated cells) for *in vitro* study.

### Chromatin immunoprecipitation (ChIP)

#### Chromatin extraction and sonication

To optimize cofactor fixation on DNA, tissues were incubated with 0.4% ChIP cross-link Gold (Diagenode) in PBS/MgCl_2_ (PBS with 1 mM MgCl_2_) for 30 min at room temperature. After washing in PBS/PIC (Protease Inhibitor Cocktail), samples were incubated with 1% formaldehyde (Sigma-Aldrich) for 5 min at room temperature. Cross-linking was stopped with 0.125 M glycine for 5 min at room temperature. After washing, samples were centrifuged for 2 min at 8000 *g* and resuspended in lysis buffer (5 mM PIPES pH 8, 85 mM KCL, 0.5% IGEPAL, PIC) in ice for 15 min. Lysates were centrifuged for 2 min, 10,000 *g* at 4° C, and pellets were incubated in lysis buffer tL1 (Diagenode) for 5 min in ice.

After adding 3 volumes of HBSS+PIC, samples were sonicated with a Bioruptor™ sonicator (Diagenode) for 10 min (10 cycles, 30 s ON/30 s OFF) at 4° C. Lysates were clarified by centrifugation (10 min at 14,000 *g*, 4° C), and supernatants were transferred to new tubes.

Before analysis, the efficiency of incubation time and sonication was checked by DNA extraction and migration on 1.5% agarose gel (Supplementary Figure 1).

#### Chromatin immunoprecipitation

Chromatin immunoprecipitation was performed using an AUTO True MicroChIP KIT (Diagenode) according to the manufacturer's protocol on an SX-8G IP-Star^®^ Compact Automated System (Diagenode). ChIP used 200 μL of sonicated chromatin and 3 μg of antibodies: anti-H3K27me3 (#C15410069, Diagenode), anti-EZH2 (#C15410039, Diagenode), anti-JMJD3 (#ab85392, Abcam) and anti-IgG for negative control (#C15410206, Diagenode). Antibody coating reaction with protein A-coated magnetic beads lasted 3 h, and the immunoprecipitation reaction 13 h at 4° C. Reverse cross-linking was carried out for 4 h at 65° C.

Immunoprecipitated DNA (IP) and total DNA (input) were purified by MicroChIP DiaPure columns (#C03040001, Diagenode) according to the manufacturer's instructions, and analyzed by real-time PCR.

#### Real-time PCR

qPCR was performed in triplicate at 25 μL final reaction volume (5 μL of IP or input, 1X de TaqMan^®^ Universal PCR Master Mix (Applied Biosystems), 400 nM for each forward and reverse primers (Sigma-Aldrich), 250 nM of probe (Applied Biosystems) (Table 2) and 4.25 μL of water) on a 7900 HT Fast Real Time PCR System (Applied Biosystems).

**Table 2:** Primers and probes used for ChIP-qPCR

Gene	Sequence primers	MGB probes
*MGMT* [7]	F: AAAGGTACGGGCCATTTGG R: GGCGCCTTCCCAGCTT	TAAGGCACAGAGCCTC
*RPS6KA2*	F: GGAGATAGACATCAGCCATCATGT R: AGCTCAAACTGGGAAGGATCTG	AAGGAGGGCTTTGAGAAG
*TRA2A*	F: CTTCGTGAAGTATGTTCTTGATATGGA R: GCCCAGTTTGCTGGTTGTAAA	CTTTGAATGGTGCCAATG
*U2AF1*	F: GAGCATGTCGTCATGGAGACA R: GGTCTGGCTAAACGTCGGTTT	TGCTCTCGGTTGCACAA

Primer and probe sequences were selected with the help of Primer Express software (ABI).

The recovery level of proteins was disclosed by the rate of IP relative to Input. The efficiency of chromatin immunoprecipitation of a particular genomic locus can be calculated from qPCR data and reported as a percentage of starting material: %(ChIP/Total Input) = 2^[(Ct(*x*%input) – log(*x*%)/log2) – Ct(ChIP)] × 100%. Ct (input) and Ct (ChIP) are threshold values obtained from the exponential phase of qPCR for the immunoprecipitated DNA sample and input sample respectively. log(*x*%)/log2 accounted for the dilution 1/*x* of the input. Before analysis, we checked the presence of proteins by the fold-enrichment on gene control *TSH2B* and *GAPDH* (Supplementary Figure 2).

### Statistical analyses

Statistical analyses were performed using R 3.0.3 software. All data followed a normal distribution, verified by four tests, namely Kolmogorov-Smirnov, Cramer-von Mises, Lilliefors and Anderson-Darling; if the distribution was abnormal, a one-parameter Box-Cox transformation was used. Data were analyzed with an ANOVA to test significant difference in gene expression average between clinicopathological groups in the patient study, between treatments and cell lines in the *in vitro* study and between genes occupancy in ChIP study. Multiple comparisons were carried out with a Tukey's *post hoc* test; statistical significance was set at *p* < 0.05.

## SUPPLEMENTARY MATERIALS FIGURES AND TABLES


