# Supplementary material for: A new metabolic gene signature in prostate cancer regulated by JMJD3 and EZH2
Source: Oncotarget. 2018 May 4;9(34):23413–25. doi: 10.18632/oncotarget.25182 (PMC5955128; doi:10.18632/oncotarget.25182)
Supplement: Supplementary file 1 [file oncotarget-09-23413-s001.pdf]

A new metabolic gene signature in prostate cancer regulated by JMJD3 and EZH2

SUPPLEMENTARY MATERIALS

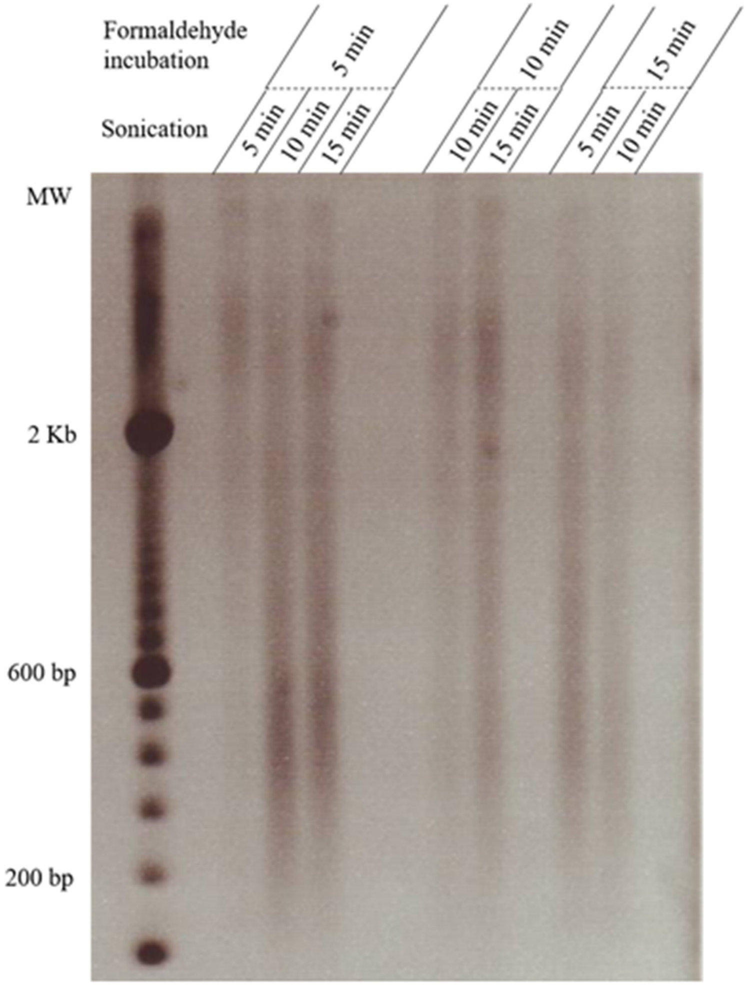

Supplementary Figure 1: DNA fragment size control. DNA molecular weight after sonication was controlled in different conditions (formaldehyde incubation time and sonication time). The ideal DNA fragment size after sonication is 200–1000 bp. This controlled has enabled us to choose the condition: 5 min of formaldehyde incubation and 10 min of sonication.

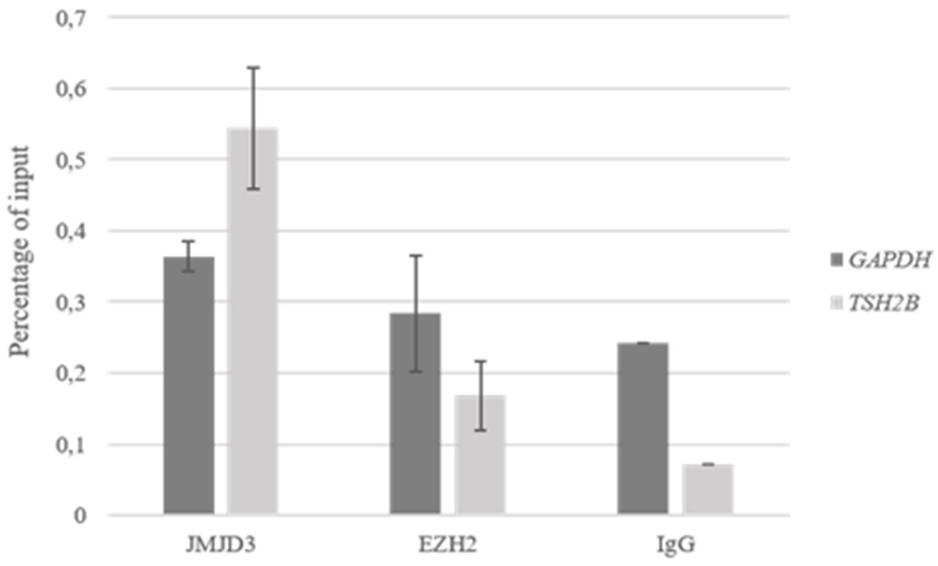

Supplementary Figure 2: ChIP control efficiency. Efficiency of ChIP was controlled by the recovery of proteins (JMJD3 and EZH2) on gene control *TSH2B* and *GAPDH*. IgG was negative control. The recovery is expressed in the average of percentage of input by protein (the relative quantity of immunoprecipitaded DNA compared to input DNA).
